# Characterizing Unaccompanied Foreign Minors: Educational Level and Length of Stay as Individual Difference Factors That Impact Academic Self-Efficacy

**DOI:** 10.3389/fpsyg.2022.780488

**Published:** 2022-02-15

**Authors:** María del Carmen Olmos-Gómez, María Dolores Pistón-Rodríguez, Ramón Chacón-Cuberos, José Javier Romero-Díaz de la Guardia, Jesús Manuel Cuevas-Rincón, Eva María Olmedo-Moreno

**Affiliations:** Department of Research Methods and Diagnosis in Education, University of Granada, Granada, Spain

**Keywords:** academic self-efficacy, multilevel analysis, inclusion, socio-economic policies, social development, vulnerable groups

## Abstract

The aim of the present study is to analyze individual differences in academic self-efficacy within a population of Unaccompanied Foreign Minors (UFM) from the European cities of Ceuta and Melilla (Spain). Variables describing educational level and length of stay were considered in a sample of 377 individuals being cared for in different youth centers. Of these, 63.4% belonged to the group who had stayed at the center for less than 9 months and 36.6% reported a length of stay of more than 9 months. The age of participants ranged between 8 and 17 years old (*M* = 14.87 years). Once the quality parameters of the instrument (academic self-efficacy) were elaborated, reliability and validity was confirmed through Confirmatory Factor Analysis (CFA) using Structural Equation Modeling (SEM) methodology. Data collection was then initiated. The results overall indicate that 87.6% of those who completed the questionnaire reported a higher level of self-efficacy with regards to working with any classmate, whilst at the same time seeing themselves as capable of achieving good marks. ANOVA results indicated significant differences with respect to educational level and length of stay. In this regard, students who had received professional training and had been at the Center for more than 9 months, were the ones who developed greater academic self-efficacy for spending more time working when tasks were judged to be difficult. The results obtained demonstrate that any intervention will be positive as long as it promotes different institutions to develop strategies that cater to a length of stay of more than 9 months and target education, academic self-efficacy, socialization and strengthening the future workforce. Such interventions can be directed through new European, Spanish or local level policies. It is clear that institutions still have a lot of work left to do.

## Introduction

In recent years there have been significant changes in Western society with the increase in migratory flow that has been taking place across Europe. Over the last two decades there has been a 200% increase in the arrival of Foreign Unaccompanied Minors (UFM) to cities that offer better basic opportunities (Menjívar and Perreira, 2019). It is a fact that this phenomenon occurs in many developed countries in this century (Sevincer et al., 2015). Application of the specific term UFM has different connotations depending on the geographic location of the country. In countries such as Italy, Switzerland, Sweden, Finland etc. UFM denominates “unaccompanied minors.” France refers to minors as “Les mineursisolés étrangers.” Spain and Belgium designate them as “Mineur étranger non-accompagné” (unaccompanied foreign minors). In this case, the term includes all of those individuals from foreign countries which do not form part of the European Union, who are younger than 18 years old and enter Spain without being accompanied by any person who can account for them (United Nations Children’s Fund [UNICEF], 2007; López de los Mozos, 2013; Tchouaga, 2015).

López de los Mozos (2013) defines UFM according to the Immigration Regulation through which the Regulation of the Organic Law 4/2000 is approved. This is the norm that is currently in force and dated within the RD 557/2011 as April 20th (Legislación Consolidada, 2011). Its deals with the rights and freedoms of immigrants in Spain and their social integration. Following its reform under Organic Law 2/2009, article 189 establishes: “The foreign minor (aged younger than 18 years) who arrives to a Spanish territory but does not come accompanied by an adult who is responsible for them, whether legally or through a fixed custom, will receive appropriate care given the risks posed that are relevant to child protection, whilst such responsible adult does not effectively take charge of the minor, just as any other immigrant who finds themself in that situation once in Spain.”

The term UFM is typically used in a political way (due to the large proliferation in aid that is perceived to be received by them) and negatively perceived by the society in which they find themselves (given that, in many cases they engage in criminal activities in order to survive). However, we cannot forget that they are children. The United Nations Convention for Children’s Rights establishes these migrant minors as “children first and foremost.” Though, this consideration has not been applied equally by member countries of the EU, with tension being evident between the need for migratory control and the requirement to apply protection policies (United Nations Children’s Fund [UNICEF], 2005; Alscher, 2017; Bravo and Santos-González, 2017).

UFM is a nomenclature associated with negative stereotypes. This brings with it an increase in negative thoughts toward these individuals, which directly affects trust, aspirations and effort within these individuals or groups. In the final instance this has implications for their learning, performance and their wellbeing. All of these aspects are impaired when social groups are stereotyped (Vecci and Želinský, 2019).

Within the reception policies of host countries, one of the most important causes of controversy is evaluating the age of the UFM. In order to obtain documentation and be able to remain in the reception country, the UFM must remain in that country for more than 2 years before turning 18. This is when they can receive documentation. Nevertheless, attending to physical factors is not enough to evaluate a minor’s age and other individual, social and cultural factors must also be considered (Garamendi et al., 2011; Cemlyn and Nye, 2012; Bravo and Santos-González, 2017; Ambrosini, 2018; Casillas-Martín et al., 2020). Thus, the determination of age is fundamental, as is belonging to a reception center, where residential care programs are developed. As a result of this, reception periods are established from a stay duration of 9 months onward, yet administrations put residential care programs into motion *via* the Individualized Education Project up until the 2 year mark. Through this, interventions for UFM are delivered *via* social integration at both a residential and school level. Before the 9 month mark, the UFM will be returned to the place they have come from should their families be located (Cachón, 2008; Laparra et al., 2014; Fokkema and De Haas, 2015; García, 2015; Millán et al., 2017). Further, a length of stay of at least 2 years and coming of age (i.e., turning 18) are not the only requirements. At the same time, the individual must be trained and integrated, or ready to be integrated, into the workforce. As already demonstrated, this has the added difficulty that their foreign origin presents an obstacle to the process of accessing the workplace (Vernby and Dancygier, 2019).

In consideration of all that has been discussed, attention to education is a crucial step toward socio-educational integration. Staying in a juvenile reception center leads to worse social adaptation, an increase in school problems and the appearance of disruptive behaviors (Millán et al., 2009; De la Herrán et al., 2018; Olmos-Gómez et al., 2020; Tomé-Fernández et al., 2020). Such behaviors may be due to various causes. These can include variables that relate to their life prior to being placed into care and are linked with the socio-family history of the minor. The role of residential care should be considered alongside the repercussions this may have on the life of young people. For instance, a lack of focused educational programs can impinge upon their social and problem solving skills (Millán et al., 2009; Martínez et al., 2010). These aforementioned social skills and problem solving skills are an inflection point for the configuration of a new social model that will generate new ways of understanding learning. Amongst other cognitive variables this model should include academic self-efficacy, which is the main predictor of academic performance (Bandura, 1995). Further, it should also strive to predict subsequent success (Bandura, 1997; Pajares and Schunk, 2001) as self-efficacy is an important cognitive mediator of competence and performance (Valiante, 2000). With regards to it favoring cognitive processes (Pintrich and De Groot, 1990) “self-efficacy is defined as the way in which a person thinks about their own capacity to organize and execute the courses of action required to achieve specific goals.” According to Bandura (1995), the events upon which this influence is exercised vary. Self-efficacy as a construct can be applied to thought processes, affective states, implementing actions, changing environmental conditions and the self-regulation of motivation” (Galleguillos-Herrera and Olmedo-Moreno, 2017; Moreno-Morilla et al., 2020).

Yusuf (2011) revealed the effects that beliefs about self-efficacy, motivation and learning strategies can have on academic performance, showing that greater self-efficacy can contribute to a significant improvement in the learning process. In a context of medical education Zheng et al. (2021) have showed that the more students believe in their ability to take actions that help them achieve academic goals, the higher their grades will be.

Given that self-efficacy is a construct that is highly correlated with several domains (performance, self-concept, effort, learning, self-confidence, understanding, motivation) in particular, it is directly related to the academic context, so the measurement of this construct linked to the cultural origin of the UFM residing in our country, gives us guidelines of their possible academic trajectories linked to their countries of reference, establishing differences with the countries in which academic self-efficacy has been studied both in general and specifically (Bandura, 1997; Stajkovic et al., 2018; Olmedo-Moreno et al., 2020). In a study with immigrant students in China, Iyer et al. (2017) propose that self-efficacy and performance in intellectual tasks, in minority social groups of low status, can improve if they are encouraged to believe in a better future for their environment, demonstrating that this belief is an important means of improving self-efficacy and educational outcomes among members of disadvantaged groups. Furthermore, in a study conducted in three schools for immigrant children in Beijing, students who identified as Beijinger were shown to have better self-efficacy, which in turn was associated with better academic performance and better relationships with their peers, compared to those who identified themselves with their rural hometown (Wang et al., 2019).

The purpose of the present research considers the principle of relativism of constructs in reference to the context of their measurement and that pointed out by Bandura (Stajkovic et al., 2018) about distancing ourselves from general measures of self-efficacy (Expósito-López et al., 2019; Olmedo-Moreno et al., 2021). It seeks to determine the potential relationships within a group of UFM who have been placed into care, and their level of academic self-efficacy as a function of educational level and duration of residence in the reception country.

## Materials and Methods

### Method

For the present investigation a quantitative study based on the social analytical-empirical research method was conducted. The study was descriptive in nature (Howell et al., 2008).

### Participants

Sample selection was made through stratified proportional sampling as a function of length of stay in the welcome country, this being less than 9 months or more than 9 months. This period is determined as the moment at which the minor has finalized the Initial Phase and Educational Development in a Temporary Relocation Centre for Vulnerable Groups. These phases last for a minimum of 1 month and are typically estimated to last 9 months depending on what the minor achieves. Requirements include amongst others, consolidating their adaptation to the educational group and the center, and outlining their educational, family and health situation. This will enable UFM’s to develop an Individualized Education Program, and fulfill the norms and objectives proposed by their institution (hygiene habits, behavior and conduct in the center). They will also be able to develop formative learning or training processes in consideration of their personal needs and resources, strengthen relationships with social support networks (school, friends, etc.), and take responsibility for their own educational program, 377 participants were selected who fulfilled the indicated characteristics. Statistical determination of the sample was considered at a confidence level of 0.01, with a precision (d) of 3% and proportion of 5%. Of the total sample, 63.4% corresponded to the group of those with a length of stay at the center that was shorter than 9 months and 36.6% had stayed for longer than 9 months. The age of participants ranged between 8 and 17 years old (*M* = 14.87 years), currently taking primary education (*n* = 38–10.1%), secondary education (*n* = 96–25.5%), vocational training (*n* = 198–52.5%) and upper-secondary (*n* = 45–11.9).

### Instruments

Each participant received the Academic Self-Efficacy scale adapted for UFM, validated by Olmedo-Moreno et al. (2020). This version is composed of 12 items grouped into three dimensions: confidence, effort and understanding. This questionnaire is based on the original 18-item scale previously validated in schoolchildren (Galleguillos-Herrera and Olmedo-Moreno, 2017). Therefore, items [I.1, I.3, I.5, I.7, I.13, and I.16] are not considered in this study, were eliminated for data analysis because they presented factorial loads lower than 0.400 (Olmedo-Moreno et al., 2020). Statements were rated on a continuum from 1 to 5 from “I never can” to “I always can.” This instrument has been validated and possesses the psychometric properties required for use. Reliability of the global scale was Cronbach alpha coefficient = 0.812 (Olmedo-Moreno et al., 2020). Specifically, in the validation study by Olmedo-Moreno et al. (2020) for this same sample, validity was assessed by means of the fit indexes of the structural model developed through the multigroup analysis with the confirmatory factor analysis, which obtained a normalized fit index (NFI = 0.906), the incremental fit index (IFI = 0.917) and the confirmatory fit index (CFI = 0.914), all of them with acceptable values. Likewise, the root means square error of approximation analysis (RMSEA = 0.052) (Olmedo-Moreno et al., 2020) obtained a more than adequate value (Jöreskog and Sörbom, 1993).

### Procedure

A pilot study was conducted to check validity. In this study, the instrument was distributed to 377 individuals (with completion taking between 30 and 60 min) in order to establish the level of comprehension from a qualitative perspective. Doubts and suggestions were also noted.

In the development of the present study, questionnaires were administered during out of hours to not interfere with the planned timetable of the center. The questionnaire was administered during the period from March 2019 to February 2020. It was approved by the directors of the participants’ centers, who collaborated with the research and advised around the optimal times to collect data. Accordingly, questionnaires were administered to the UFM. A researcher was present throughout distribution and completion so that any questions relating to comprehension and completion of the task could be answered. Once agreement was secured from the director of the centers, questionnaires were administered to small groups of children. Questionnaires were written in the Amazing language (as appropriate) in order to facilitate better comprehension. Administration of each questionnaire lasted 25 min. Due to the linguistic and cross-border characteristics of the city of Melilla and Ceuta, the questionnaire was translated into the mother tongue (Amazing or Dariya) of the population living in the city. The Amazing and Dariya dialect is only spoken and has no written transcription. For this reason, it was necessary to translate the questionnaire into the mother dialect of this population (Amazing). A registry of interviews was produced *via* audio recordings in this dialect. Ten postgraduate students were present in order to provide support and facilitate understanding of questions. Participants were informed that all of the collected information would only be used for the purposes of scientific research and that anonymity of participants would be preserved. Participants were not informed of the purpose of the study in order to avoid insincere responses being given and to reduce as much as possible the effects of social desirability.

Melilla is a city located in the north eastern part of Magreb, known as Rif, and has a population of approximately 83,679 inhabitants. It is the city with the highest birth rate in Spain, together with one of the lowest mortality rates.

Ceuta is a city situated on the Eastern side of the Tingitana peninsula, on the African shore of the Strait of Gibraltar. It has a population of 84,959 inhabitants. Its land border separates the Moroccan prefectures of Fahs Anjra and M’Diq-Fnideq, both of which belong to the Tánger-Tetuán-Alhucemas region.

### Data Analysis

For the purpose of data analysis, content analysis was employed to analyze the qualitative questions. In addition, quantitative data were analyzed according to descriptive statistics and estimations of internal consistency. This was conducted using SPSS 24.0.

Data were tested in order to determine the type of statistical procedure to be adopted (parametric or non-parametric tests). Firstly, the normality of the data was checked, reviewing the values of skewness and kurtosis of the different items that make up the questionnaire. In this case, no high dispersion was obtained, assuming normality in the data and opting for parametric tests. Secondly, parametric tests were used as the assumption of homogeneity of variance was satisfied through Levene’s test and the sample size was sufficiently large (Andréu, 2011).

ANOVA was used employing a multi-level design for multiple comparisons of various factor levels. This was conducted to evaluate differences between participating groups according to their level of academic self-efficacy as a function of their educational level and length of residence in the reception country. Two-way ANOVA was used for each of the questionnaire items to determine their individual scores. The same analysis is also performed for the three dimensions of self-efficacy set by Olmedo-Moreno et al. (2020). It should be noted that *post hoc* tests (Duncan Test) were applied in order to specify statistically significant inter-group differences.

## Results

Table 1, showing means and standard deviations for self-efficacy as a function of educational level and length of stay, demonstrates that children who undertake professional training modules have a higher level of self-efficacy with respect to certain processes. This included working with any classmate and achieving good marks, completing a task successfully, understanding the teaching of any teacher and studying independently in order to get good marks. Those undertaking a baccalaureate considered their self-efficacy to be highest in relation to getting good marks for difficult tasks. With regards to those children who had resided in care for more than 9 months, these reported greater self-efficacy when it came to understanding the teaching of any teacher and studying independently in order to get good marks.

**TABLE 1:** Means (M) and standard deviations (SD) for self-efficacy as a function of educational level (E.L) and length of stay.

		Less than 9 months		More than 9 months		Total	
	E.L.	*M*	*SD*	*M*	*SD*	*M*	*SD*
I.2. *Hacer una tarea con éxito* [Complete a task successfully]	P.E	3.17***^*d*^***	1.378	3.65	1.471	3.46	1.448
	S.E	3.78	1.343	3.98	1.141	3.89	1.237
	B	3.67	1.211	3.85	1.214	3.77	1.166
	V.T	4.19***^*d*^***	1.131	4.32	0.944	4.24	1.055
	Total	3.71	1.336	3.90	1.272	3.81	1.305
I.4. *Realizar bien cualquier tarea que me den* [Carry out well any task given to me]	P.E	3.57	1.382	3.76	1.433	3.69	1.410
	S.E	4.06	1.298	3.77	1.134	3.91	1.215
	B	4.33	0.816	3.28	1.253	3.77	1.166
	V.T	4.21	1.094	4.14	1.044	4.19	1.067
	Total	3.97	1.268	3.81	1.257	3.89	1.263
I.6. *Esforzarme más que mis compañeros para tener éxito en las tareas* [Work harder than my classmates to succeed in tasks]	P.E	3.88	1.329	4.17	1.251	4.06	1.285
	S.E	3.78	1.476	3.87	1.389	3.83	1.424
	B	3.67	1.506	3.85	1.463	3.77	1.423
	V.T	4.19	1.292	4.17	1.188	4.19	1.243
	Total	3.93	1.376	4.05	1.298	3.99	1.335
I.8. *Organizar mi tiempo para hacer todo lo que los profesores piden* [Organize my time to complete everything that the teachers set]	P.E	3.71	1.312	4.36	1.140	4.10	1.247
	S.E	4.04	1.274	3.80	1.328	3.92	1.303
	B	4.00	1.265	4.14	1.069	4.08	1.115
	V.T	4.33	1.028	4.17	0.983	4.27	1.006
	Total	4.03	1.227	4.11	1.200	4.07	1.212
I.9. *Sacar buenas notas en pruebas difíciles* [Get good marks on difficult tests]	P.E	3.40	1.251	3.65	1.297	3.55	1.278
	S.E	3.35***^*d*^***	1.300	3.61	1.097	3.49	1.197
	B	4.00***^*d*^***	0.632	4.14	0.690	4.08	0.641
	V.T	3.95	1.058	4.03	0.881	3.99	0.985
	Total	3.58	1.216	3.72	1.141	3.66	1.178
I.10. *Trabajar más tiempo cuando tengo tareas difíciles* [Work for longer when I have difficult tasks]	P.E	3.48***^*d*^***	1.234	4.26	1.080	3.95	1.204
	S.E	4.16	1.161	3.89	1.304	4.02	1.242
	B	3.50	1.643	4.14	0.690	3.85	1.214
	V.T	4.43***^*d*^***	0.859	4.42	0.878	4.43	0.861
	Total	4.01	1.183	4.15	1.134	4.09	1.158
I.11. *Esforzarme más para resolver tareas difíciles* [Try harder to solve difficult tasks]	P.E	3.67	1.408	4.22	1.023	4.00	1.217
	S.E	4.24	1.031	3.92	1.251	4.08	1.160
	B	4.50	0.548	3.71	1.112	4.08	0.954
	V.T	4.43	0.991	4.35	0.951	4.40	0.969
	Total	4.14	1.168	4.11	1.110	4.13	1.136
I.12. *Repetir una tarea hasta lograr hacerla bien* [Repeat a task until I manage to do it well]	P.E	3.79	1.298	4.28	1.262	4.09	1.294
	S.E	4.33	1.144	4.33	1.023	4.33	1.075
	B	5.00	0.000	4.00	1.290	4.46	1.050
	V.T	4.55	0.916	4.28	1.083	4.44	0.987
	Total	4.26	1.151	4.29	1.139	4.28	1.143
I.14. *Esforzarme en trabajar bien con otros compañeros en tareas en grupo* [Strive to work well with other classmates on group tasks]	P.E	4.21	1.001	4.36	1.195	4.30	1.119
	S.E	4.22	1.195	4.45	0.803	4.35	1.005
	B	4.67	0.516	4.57	0.534	4.62	0.506
	V.T	4.36	1.008	4.35	1.129	4.36	1.050
	Total	4.28	1.057	4.40	1.023	4.35	1.040
I.15. *Estudiar solo para sacar buenas notas* [Study independently in order to get good marks]	P.E	3.50***^*d*^***	1.469	3.98	1.263	3.79	1.364
	S.E	3.63***^*d*^***	1.185	4.07***^*d*^***	0.942	3.87	1.079
	B	3.67	1.366	3.85	1.069	3.77	1.166
	V.T	4.26***^*d*^***	1.083	4.28	0.896	4.27	1.006
	Total	3.78	1.284	4.06	1.079	3.93	1.187
I.17. *Entender lo que dice el profesor cuando hay ruido en clase* [Understand what the teacher says when there is noise in class]	P.E	2.69	1.473	2.49	1.574	2.57	1.531
	S.E	2.67	1.560	2.70	1.647	2.69	1.600
	B	3.33	1.211	2.28	1.704	2.77	1.536
	V.T	2.67	1.648	2.75	1.506	2.70	1.582
	Total	2.71	1.539	2.60	1.585	2.65	1.562
I.18.*Estudiar primero y hacer otras cosas luego*[Study first and do other things later]	P.E	2.71	1.391	3.48	1.661	2.61	1.448
	S.E	2.56	1.411	3.73	1.545	2.66	1.598
	B	3.03	1.132	3.17	1.698	2.79	1.497
	V.T	2.78	1.538	3.49	1.602	2.83	1.496
	Total	2.79	1.489	3.55	1.589	2.72	1.542

*P.E, primary education; S.E, secondary education; V.T, vocational training; B, Baccalaureate. ***^*d*^***, Duncan’s test (*Post hoc*) for differences inter-groups.*

Table 2 shows multivariate ANOVA (MANOVA) results produced for the variables describing the length of time spent in Spain and educational level, and the interactions between them. Effect size was calculated using η^2^. Initially, multivariate analysis indicated significant differences and moderate effect sizes with regards to educational level (*F* = 2.971, *p* < 0.001, and η^2^ = 0.157), significant differences with regards to the length of time spent in Spain (*F* = 1.310, *p* < 0.001, and η^2^ = 0.076) and a significant interaction between the length of time spent in Spain and educational level (*F* = 905.071, *p* < 0.001 and η^2^ = 0.183).

**TABLE 2:** Multivariate ANOVA (MANOVA) results and effect sizes (η^2^) for academic self-efficacy, the length of time spent in Spain and educational level.

	Educational level (E.L.)			Length of stay in Spain (L.S)			E.L. x L.S		
	F	*P*	η^2^	F	*p*	η^2^	F	*p*	η^2^
I.2. *Hacer una tarea con éxito* [Complete a task successfully]	5.471	0.001	0.054	2.443	0.119	0.008	0.070	0.976	0.001
I.4. *Realizar bien cualquier tarea que me den* [Complete well any task given to me]	2.276	0.080	0.023	0.189	0.664	0.001	1.557	0.200	0.016
I.6. *Esforzarme más que mis compañeros para tener éxito en las tareas* [Work harder than my classmates to succeed in tasks]	1.206	0.308	0.012	0.778	0.378	0.003	0.134	0.940	0.001
I.8. *Organizar mi tiempo para hacer todo lo que los profesores piden* [Organize my time to complete everything that the teachers set]	1.273	0.284	0.013	0.880	0.349	0.003	2.636	0.050	0.027
I.9. *Sacar buenas notas en pruebas difíciles* [Get good marks on difficult tests]	3.413	0.018	0.035	1.864	0.173	0.006	0.090	0.966	0.001
I.10. *Trabajar más tiempo cuando tengo tareas difíciles* [Work for longer when I have difficult tasks]	2.976	0.032	0.030	2.990	0.085	0.010	4.029	0.008	0.041
I.12. *Repetir una tarea hasta lograr hacerla bien* [Repeat a task until I manage to do it well]	1.700	0.167	0.018	0.494	0.483	0.002	2.748	0.043	0.028
I.13. *Entender bien la idea que explica el profesor o el libro* [Understand well the idea explained by the teacher or in a book]	1.283	0.281	0.013	3.253	0.072	0.011	0.957	0.413	0.010
I.14.*Esforzarme en trabajar bien con otros compañeros en tareas en grupo* [Strive to work well with other classmates on group tasks]	0.344	0.794	0.004	0.576	0.448	0.002	0.260	0.854	0.003
I.15. *Estudiar solo para sacar buenas notas* [Study independently in order to get good marks]	2.679	0.047	0.027	7.022	0.009	0.024	0.625	0.599	0.007
I.17. *Entender lo que dice el profesor cuando hay ruido en clase* [Understand what the teacher says when there is noise in class]	0.157	0.925	0.002	0.461	0.498	0.002	0.633	0.594	0.007
I.18.*Estudiar primero y hacer otras cosas luego* [Study first and do other things later]	0.978	0.403	0.010	0.029	0.866	0.001	0.925	0.382	0.011

On the one hand, fit of the ANOVA results to the observed data revealed tighter associations when educational level and length of stay were considered. Students with professional training who had resided in Spain for longer than 9 months presented higher academic self-efficacy, specifically for aspects describing working for longer when tasks were difficult (*p* = 0.008) and repeating tasks in order to manage to complete it well (*p* = 0.043).

The results indicated significant differences and large effect sizes with respect to educational level, which demonstrated its importance in the processes of academic development of UFMs. The sample size or the proportion of variance explained (Richardson, 2011; Badenes et al., 2018) indicated that, with respect to educational level, more than 15% (η^2^ = 0.157) of the differences found could be attributed to the effects of differences in educational level, since the studies they access once in reception and which are those referred to in this research with respect to the studies of those coming from their place of origin, provide foundations for their future academic development. Likewise, the results obtained showed significant differences with respect to the length of time spent in Spain and the sample size (Richardson, 2011; Badenes et al., 2018) indicated that 7.6% (η2 = 0.076) of the differences found could be attributed to the effect of the length of time spent in Spain, since the studies they access once in reception and which are those referred to in this research with respect to the studies of those who come from their place of origin, provide a basis for their future academic development. As for the sample size in relation to the interaction between academic level and the length of time spent in Spain, the value of which was about one-fifth. In social terms and according to the study, this is a value to consider since 18% (η^2^ = 0.183) of the differences found were attributed to the effect of the difference in relation to this interaction.

Next, three univariate linear models are run in order to test the differences between the dimensions of self-efficacy according to educational level and length of residence -two routes- (Table 3). Firstly, the test of equality of variances through Levene’s Test reflected a value of 0.379 for factor 1 (confidence), 0.065 for factor 2 (effort) and 0.238 for factor 3 (understanding), assuming the acceptance of the null hypothesis and proceeding with the execution of the three models.

**TABLE 3:** Model fit by dimension.

Dimension	Origin	Sum of squares	DF	Quadratic mean	*F*-Test	Sig.	η^2^	*R*^2^ (R adjusted)
Confidence	Corrected model	3.288	5	0.658	1.574	0.168	0.030	0.030 (0.011)
	Intersection	3,695.968	1	3,695.968	8,843.006	0.000	0.972	
	Time	0.015	1	0.015	0.035	0.852	0.000	
	Level	2.737	2	1.368	3.274	0.039	0.025	
	Time * Level	0.359	2	0.180	0.430	0.651	0.003	
	Error	106.996	256	0.418	–	–	–	
	Total	4,114.438	262	–	–	–	–	
	Total corrected	110.285	261	–	–	–	–	
Effort	Corrected model	3.729	5	0.746	1.233	0.294	0.024	0.024 (0.004)
	Intersection	3,951.356	1	3,951.356	6,529.255	0.000	0.962	
	Time	0.094	1	0.094	0.156	0.694	0.001	
	Level	1.516	2	0.758	1.253	0.287	0.010	
	Time * Level	0.957	2	0.478	0.790	0.455	0.006	
	Error	154.925	256	0.605	–	–	–	
	Total	4,458.000	262	–	–	–		
	Total corrected	158.655	261	–	–	–	–	
Understanding	Corrected model	4.937	5	0.987	1.467	0.201	0.028	0.028 (0.009)
	Intersection	3,000.185	1	3,000.185	4,457.039	0.000	0.946	
	Time	4.494	1	4.494	6.677	0.010	0.025	
	Level	0.610	2	0.305	0.453	0.636	0.004	
	Time * Level	0.103	2	0.051	0.076	0.927	0.001	
	Error	172.322	256	0.673	–	–	–	
	Total	3,360.563	262	–	–	–	–	
	Total corrected	177.259	261	–	–	–	–	

In this case, the data provided by the three models are discrete, reflecting an *R*^2^-value of 0.030 for confidence as the dependent variable, 0.024 for effort and 0.028 for understanding as the dependent variable. Likewise, statistically significant differences are only observed for educational level (p = 0.039) when confidence is placed as the dependent variable and for time (*p* = 0.010) when understanding is placed as the dependent variable; obtaining in both cases mild-moderate effect sizes (η^2^ = 0.025).

**FIGURE 1:**
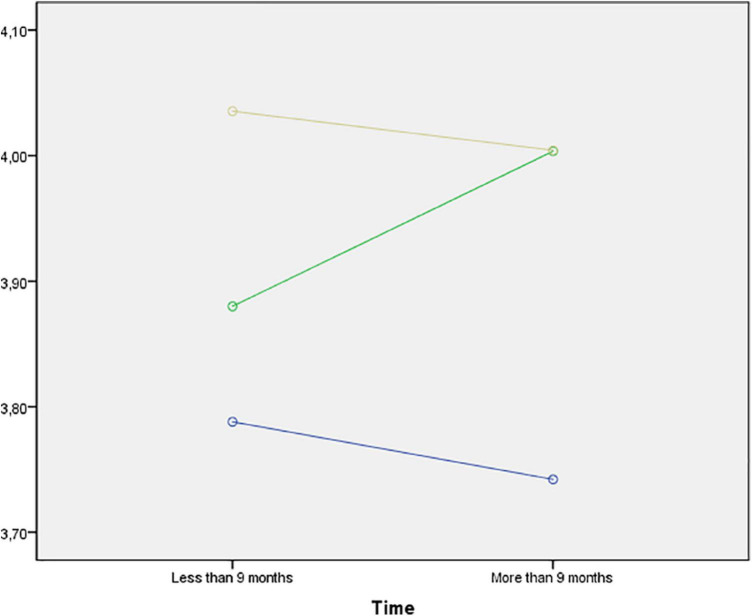
Mean values of confidence (factor 1) by educational level and time of residence. Note: Blue (Primary Education; Green (Secondary Education); Yellow (Baccalaureate—Vocational Training).

**FIGURE 2:**
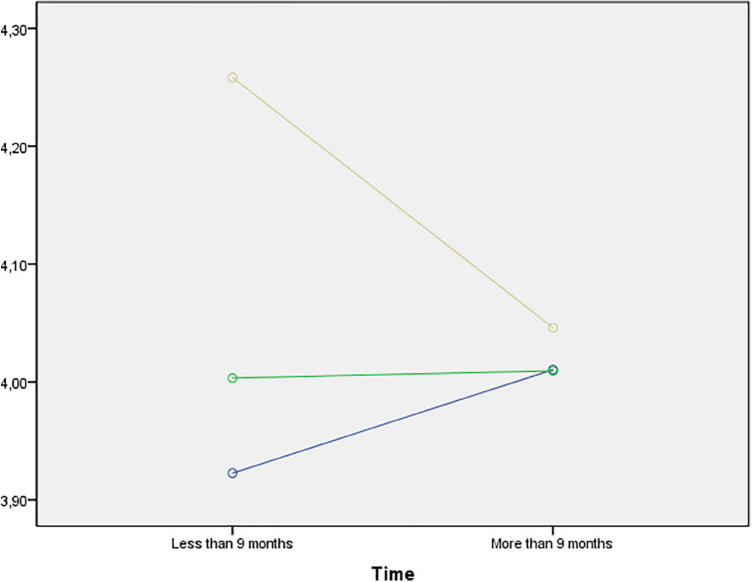
Mean values of effort (factor 2) by educational level and length of residence. Note: Blue (Primary Education; Green (Secondary Education); Yellow (Baccalaureate—Vocational Training).

**FIGURE 3:**
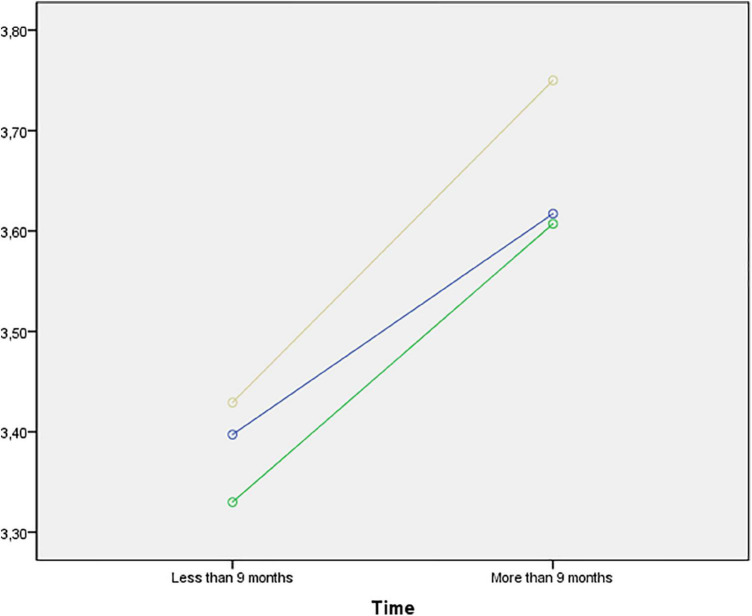
Mean values of Understanding (factor 3) by educational level and time of residence. Note: Blue (Primary Education; Green (Secondary Education); Yellow (Baccalaureate—Vocational Training).

Table 4 shows the mean values for each category of the efficacy dimensions. The model revealed statistically significant differences for educational level (*p* = 0.039) in the confidence dimension. Specifically, these are observed in the inter-group comparison, with elementary education subjects showing the lowest levels (3.78 ± 0.67 vs. 3.88 ± 0.73 vs. 4.03 ± 0.57), as shown in Figure 1. Although no statistically significant differences were found when placing effort (factor 2) as the dependent variable, Figure 2 shows the mean values considering educational level and length of residence in the different educational stages. On the other hand, statistically significant differences were also observed for time of residence in the comprehension dimension (*p* = 0.010), improving in all educational stages in those young people who had been living in Spain for longer: elementary education (3.39 ± 0.93 vs. 3.61 vs. 0.74); secondary education (3.33 ± 0.83 vs. 3.60 ± 0.84); baccalaureate (3.42 ± 0.75 vs. 3.75 ± 0.71), as shown in Figure 3.

**TABLE 4:** Descriptive variables of the dimensions according to time and educational level.

Dimension	Time	Level	Mean	SD
Confidence	<9 months	P.E	3.78***^d^***	0.67
		S.E	3.88	0.73
		Baccalaure.-VT	4.03***^d^***	0.57
	>9 months	P.E	3.74	0.66
		S.E	4.00	0.64
		Baccalaure.-VT	4.00	0.53

**Dimension**	**Time**	**Level**	**Mean**	**SD**

Effort	<9 months	P.E	3.92	0.87
		S.E	4.00	0.79
		Baccalaure.-VT	4.25	0.70
	>9 months	P.E	4.01	0.86
		S.E.	4.00	0.77
		Baccalaure.-VT	4.04	0.54

**Dimension**	**Time**	**Level**	**Mean**	**SD**

Understanding	<9 months	P.E	3.39***^d^***	0.93
		S.E	3.33***^d^***	0.83
		Baccalaure.-VT	3.42***^d^***	0.75
	>9 months	P.E	3.61***^d^***	0.74
		S.E	3.60***^d^***	0.84
		Baccalaure.-VT	3.75***^d^***	0.71

*^d^, Duncan’s test (Post hoc) for differences inter-groups.*

## Discussion

The present study analyzed the interaction between length of stay in Spain and educational level in terms of the self-concept of a sample of UFM from the cities of Ceuta and Melilla, in the south of Spain (Europe). 1 Spain is one of the border countries of the European Union that, according to the Spanish Interior Ministry, registered 3,997 minors safe-guarded or supervised by protection services in 2016. Official statistics indicate that from 2014 up until the middle of 2018, a total of 16,379 immigrant minors also arrived into the country. This number would probably be even higher if it had been possible to register minors who live on the streets and have never resided within an institutional center (Keles et al., 2018). The State Attorney General’s Office describes the evolution of the arrival of these minors as “highly concerning,” considering that it is impossible to know how many have arrived undercover or illegally at the borders of Ceuta and Melilla, or to the Spanish coastal regions.

These settings are therefore of great interest for sociological, anthropological and educational research. According to the Spanish Institute for National Statistics (Instituto Nacional de estadística [INE], 2019), despite both being two of the smallest cities in size, they have taken in a fifth of the country’s overall UFM population between 2014 and 2019. A total of 915 UFM were counted in the first 9 months of 2019.

To this end, it is important to highlight some significant data that should be more deeply analyzed and examined in later studies. These data refer to outcomes obtained for the self-efficacy of children according to their educational level. Data were more significant in relation to the following items; The data that most stood out related to students with professional training and a baccalaureate qualification. These individuals also made up a large percentage of the overall sample. When in professional training, UFM have preferential access to studies due to them being a vulnerable group at risk of social exclusion. A total of 87.6% of these individuals reported a higher level of self-efficacy with regards to working with any classmate. This suggests that socio-educational projects improve socialization. The responses intimate the importance of education as a key element in the social and educational integration of vulnerable minors (Spencer and Delvino, 2019). Further, respondents viewed themselves as capable of achieving good marks, 83.8% felt they could complete a task successfully, 88.6% understood the teaching of any teacher and 85.2% believed they could study independently in order to get good marks. Of those in the baccalaureate group, 80% considered their self-efficacy to be high in terms of getting good marks on difficult tests. This has also been corroborated by Crombach et al. (2014) who previously linked a higher educational level with better social integration. With respect to children who had resided in Spain for more than 9 months, 83% reported higher self-efficacy toward understanding the teaching of any teacher and 85.8% toward studying independently in order to get good marks. Given that the intention of these minors is to remain in Spain and acquire documentation, their self-efficacy is highly focused on taking advantage of the educational opportunity offered to them and investing effort into their training. As has been stated by Larrinaga et al. (2018) their objective is to “search for a better life” and this implies that what they really want is to work. This is due to the fact that although international studies exist which have sought to better understand important aspects of the vital development of these children and the policies available to support their integration (Engebrigtsen, 2003; Wallin and Ahlström, 2005; Derluyn and Broekaert, 2008; Hopkins and Hill, 2008; NíRaghallaigh and Gilligan, 2010), research into the influence of self-efficacy on social integration is lacking. Wallin and Ahlström (2005) examined the topic of education as one of the key cogs in the process of UFM integration, although they did not give specific information around the strategies that would bring about this integration. This is also the same in Spain, where studies based on socio-educational strategies for these minors are non-existent. From this perspective, the importance of the present study is clear. We refer back to the highly relevant finding that within the 377 individuals who responded to the survey 25.2% had Professional Training, whilst the most commonly reported level of schooling was Primary Education at 31.8% and Compulsory Secondary Education at 31.8%. An insignificant proportion of 5.3% reported having a Baccalaureate. This sets them far apart from native teenagers.

Our results show that staying in the reception center for more than 9 months promotes better academic self-efficacy, improving the understanding dimension in all educational stages in those young people who have been living in Spain for longer, with children of younger age and educational level showing lower levels of confidence, because confidence is directly related to the vital development of these children, as they are still in the process of integration in their new context, and do not yet know how to act on them. In contrast, children with a higher level of education have higher scores in terms of autonomous skills and personal initiative. Likewise, and based on the results obtained by dimensions, statistically significant differences are only observed for educational level (*p* = 0.039) when confidence is placed as the dependent variable, highlighting within this dimension the term “ability,” which gives importance to the assessment that the UFM makes about their competence for the effectiveness of their study time and perform tasks by developing learning strategies (Baas et al., 2015). Linked to the length of residence, statistically significant differences (*p* = 0.010) are obtained when understanding is taken as the dependent variable, the main items of which included self-esteem-enhancing terms such as “success” of vital importance for their personal and academic development. According to Núñez et al. (2015) and Zee and Koomen (2016), more time spent in school leads to higher academic performance, which could explain these results. With respect to both cases (confidence and comprehension) the effect sizes have been mild-moderate (η^2^ = 0.025).

From the data obtained we can confirm that the protocol for good practice employed by Spain (as with other European countries) in order to handle immigration issues, enables better integration of children and higher self-efficacy. This is made possible through the opportunities that it offers to UFM over a 9-month timeframe, leading them to exert more effort in order to obtain a good education. This protocol for good practice establishes that once in a country, minors become the responsibility of the autonomous communities or cities that regulate their legal status (Legislación Consolidada, 2001). It guarantees that UFM start to reside in institutional care centers within the host cities and strengthens education provision in related institutions. Here they also receive nutrition, housing, medical assistance and psychosocial support.

ANOVA test outcomes demonstrated significant differences and various effect sizes in the frequency of responses given by participants with regards to academic self-efficacy as a function of educational level and length of stay in the reception city. Outcomes highlight a significant (*F* = 905.071, *p* < 0.001 and η^2^ = 183) interaction between the time spent in Spain and educational level. Those who had resided in Spain for longer than 9 months and reported professional training obtained higher self-efficacy scores and produced a large effect size for eta-squared.

## Conclusion

The present research concludes as its main outcome that when the stay of UFM at reception centers exceeds 9 months, better academic self-efficacy is promoted, especially within students undertaking professional training. Individuals with this educational level obtained higher scores for skills pertaining to autonomy and personal initiative. These individuals became more aware of their knowledge, competence and environment, and were, consequently, able to take action over them. They are more capable of identifying opportunities, and developing personal and group projects. Finally, having a higher educational level provides help to the individual so they can find their place in the world. These are consequences of the situation of the UFM themselves. Findings conclude that, while significant differences do not exist between lengths of stay when considered as an individual level variable, interactional effects are found with educational level.

Another conclusion that can be extracted from the multi-level study is that significant differences exist between the various educational levels when they are considered as independent variables. Further, the largest interactional mean was produced for the level of Professional Training and Baccalaureate. Spanish studies based on socio-educational strategies for these minors are non-existent. From this perspective, the importance of the present study is clear given that, with regards to the schooling of the 377 individuals who responded to the survey, most relevant data comes from vocational training. This is at least partly due to the fact that UFM need professional training for their incorporation into the workforce and in order to secure residency papers in Europe. At this age they are more conscious of their future status and, thus, their academic self-efficacy is higher. It is also possible that more practical and less abstract material is more apt for these minors, given that their personal situation may have conditioned them to better appreciate such material. Indeed, this is one of the educational promotion strategies proposed as one of the main pillars of the process of UFM integration.

The first limitation of the present study is that we cannot know whether in the transitional process, children had been schooled in their place of origin, as only 6.1% responded to this question. Secondly, it will be necessary to broaden the sample in order to include UFM’s from other regions of Spain with the aim of obtaining significant results in the data collected from the questionnaire. This would also enable comparisons to be made with the policies employed in other regions.

Amongst the limitations identified in the present study, a key challenge was the difficulty of accessing the sample, although the time required to collect data was found to be reasonable. From this it is concluded that the results of the study allow multi-dimensional analysis to be conducted. We highlight the need to carry out new studies capable of providing evidence for the effects of other identification variables such as gender, origin, etc. This will lead to better understanding of academic self-efficacy in UFM’s.

From the present research it can be deduced that any intervention, whether delivered through European, Spanish or local level policies, will be positive as long as it targets those at centers for more than 9 months and is delivered through education, academic self-efficacy, socialization and strengthening the future workforce. Other motivations such as fear of the “pull effect” or electoral politics should be avoided.

## Data Availability Statement

The data presented in this study are available on request from the Principal Researcher: EO-M, emolmedo@ugr.es.

## Ethics Statement

This study was conducted according to the guidelines of the Declaration of Helsinki, and approved by the Ethical Committee for Psycho-educational Research of the University of Granada (201-300 Academic Ranking of World Universities, Shanghai, 2018), being approved with code 742/CEIH72018. Written informed consent to participate in this study was provided by the participants’ legal guardian/next of kin.

## Author Contributions

MO-G, EO-M, and MP-R conceived the hypothesis of this study. MO-G participated in data collection and supervision. RC-C, MO-G, and JC-R analyzed the data. MO-G and RC-C participated in writing—review and editing. MO-G, JR-D, and JC-R: wrote the manuscript, with significant input from MO-G. All authors contributed to data interpretation of the statistical analysis, wrote the manuscript with significant input, contributed to the data analysis interpretation, and read and approved the final manuscript.

## Conflict of Interest

The authors declare that the research was conducted in the absence of any commercial or financial relationships that could be construed as a potential conflict of interest.

## Publisher’s Note

All claims expressed in this article are solely those of the authors and do not necessarily represent those of their affiliated organizations, or those of the publisher, the editors and the reviewers. Any product that may be evaluated in this article, or claim that may be made by its manufacturer, is not guaranteed or endorsed by the publisher.
